# P-2159. Pneumonia in Pediatric HSCT Recipients: A Multicentric Prospective Cohort Analysis of Mortality

**DOI:** 10.1093/ofid/ofaf695.2322

**Published:** 2026-01-11

**Authors:** Silvio R Araujo, Mario A Bustos, Orlando Garcia, Miguel A Luengas-Monroy, Kevin Gonzalez, Martha Avilés-Robles, Alejandro Diaz Diaz, Gabriela Ensinck, Dennise Vaquera, Abiel H Mascareñas, José I Castillo, Rodrigo Garcia, Jose F Vallejo, Paola Marsela Pérez Camacho, Jaime Alberto Patiño Niño, Lina M Sandoval-Calle, Viviana Stefanie Martínez Osorio, Juan Sebastián Navarro Yaruro, Mauricio Chaparro Alzogaray, Almudena Laris Gonzalez, Andrea Restrepo Gouzy, Silvina Lobertti, Santiago López Papucci, Romina Valenzuela, Carlos A Portilla, Jorge Buitrago, Erika Cantor, Oscar Ramirez, María Elena Santolaya, Eduardo Lopez-Medina

**Affiliations:** Universidad del Valle, Cali, Valle del Cauca, Colombia; Universidad del Valle, Cali, Colombia, Cali, Valle del Cauca, Colombia; Universidad del Valle, Cali, Valle del Cauca, Colombia; Fundación Hospital Pediátrico la Misericordia, Bogota, Distrito Capital de Bogota, Colombia; Centro Médico ABC, Ciudad de Mexico, Estado de México, Mexico; Hospital Infantil de México Federico Gómez, Ciudad de Mexico, Estado de México, Mexico; Hospital General de Medellin, Medellin, Antioquia, Colombia; Hospital de Niños Victor Vilela, Rosario, Santa Fe, Argentina; Hospital de Niños Victor J. Vilela, Rosario, Santa Fe, Argentina; Hospital Universitario Dr. José Eleuterio González, Monterrey, Estado de México, Mexico; Christus Muguerza Sistemas de Salud, S.A de C.V, Monterrey, Nuevo Leon, Mexico; Hospital Universitario Dr. José Eleuterio González, Monterrey, Estado de México, Mexico; Clinica Imbanaco and Universidad del Valle, Cali, Valle del Cauca, Colombia; Fundación Valle del Lili, Cali, Valle del Cauca, Colombia; Fundación Valle del Lili, Cali, Valle del Cauca, Colombia; Fundación Valle del Lili, Cali, Valle del Cauca, Colombia; Fundación Hospital Pediátrico la Misericordia, Bogota, Distrito Capital de Bogota, Colombia; Fundación Hospital Pediátrico la Misericordia, Bogota, Distrito Capital de Bogota, Colombia; Fundación Hospital Pediátrico la Misericordia, Bogota, Distrito Capital de Bogota, Colombia; Hospital Infantil de Mexico Federico Gomez, London, England, United Kingdom; Hospital Pablo Tobón Uribe, Medellin, Antioquia, Colombia; Hospital de Niños Victor J. Vilela, Rosario, Santa Fe, Argentina; Hospital de Niños Victor J. Vilela, Rosario, Santa Fe, Argentina; Universidad de Chile, Providencia, Region Metropolitana, Chile; Clinica Imbanaco Grupo Quironsalud, Univerdidad del Valle, POHEMA Foundation, Universidad del Valle, Cali, Valle del Cauca, Colombia; Clinica Imbanaco Grupo Quironsalud, Cali, Valle del Cauca, Colombia; Pontificia Universidad Javeriana, Centro de Estudios en Infectolgia Pediatrica CEIP, Cali, Valle del Cauca, Colombia; Clinica Imbanaco Grupo Quironsalud, Cali, Valle del Cauca, Colombia; Universidad de Chile, Providencia, Region Metropolitana, Chile; Centro de Estudios en Infectología Pediátrica CEIP, Departamento de Pediatría, Universidad del Valle, Clínica Imbanaco, Grupo Quironsalud, Colombia., Cali, Valle del Cauca, Colombia

## Abstract

**Background:**

Infections are common complications in pediatric allogeneic hematopoietic stem cell transplantation (HSCT), with pneumonia often leading to poor outcomes. Data on pediatric patients with pneumonia post-HSCT are limited. This study describes the clinical characteristics and outcomes of affected patients, including those who survived and those who did not.

**Figure:**
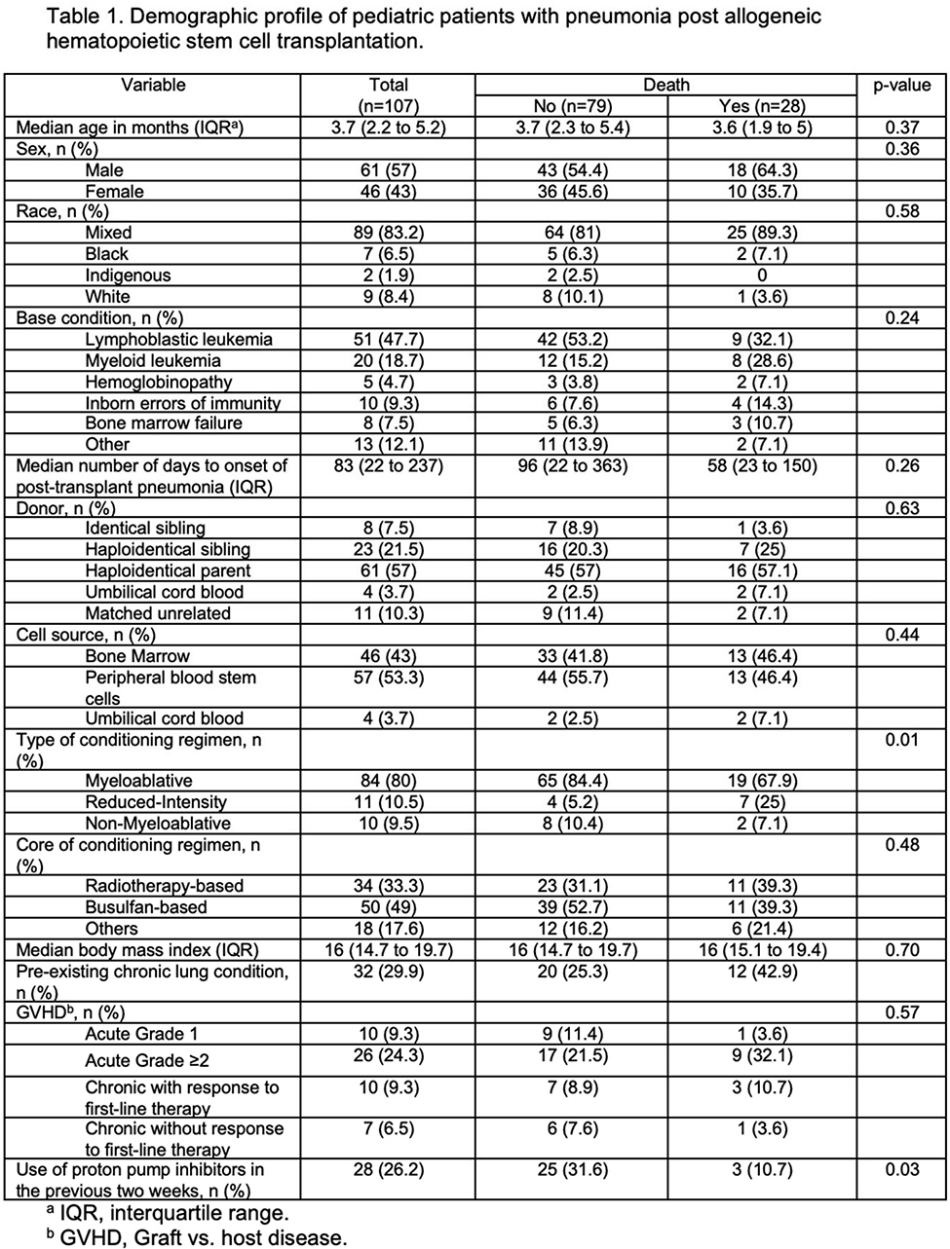

**Figure:**
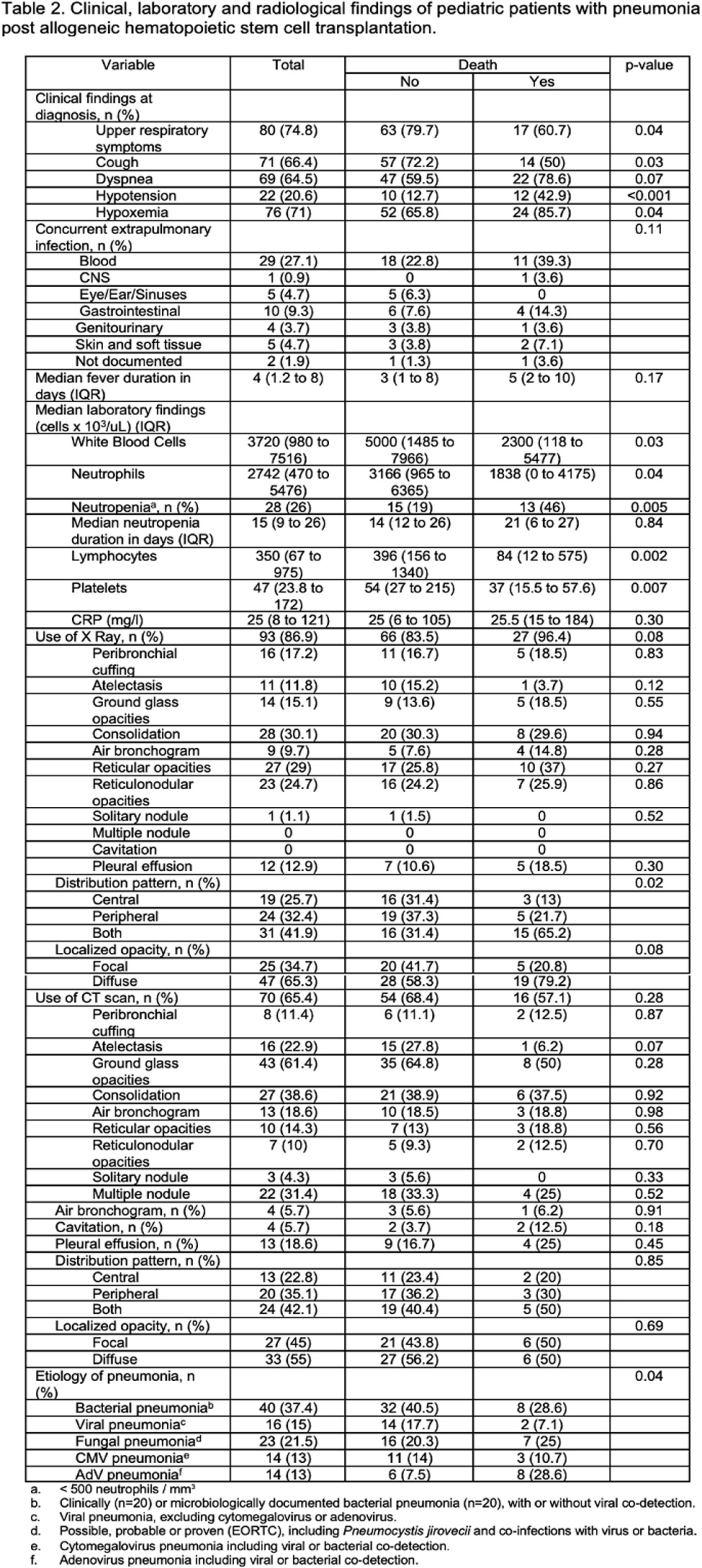

**Methods:**

In this prospective cohort study, pediatric patients with pneumonia following HSCT were enrolled between July 2022 and February 2025 across nine HSCT units in Colombia, Mexico, Argentina, and Chile. Cases were identified through active surveillance. Pneumonia was defined as the presence of fever and radiologic infiltrates. The primary outcome was pneumonia-related mortality, as determined by site investigators. Patients were followed until symptom resolution or death.

**Figure:**
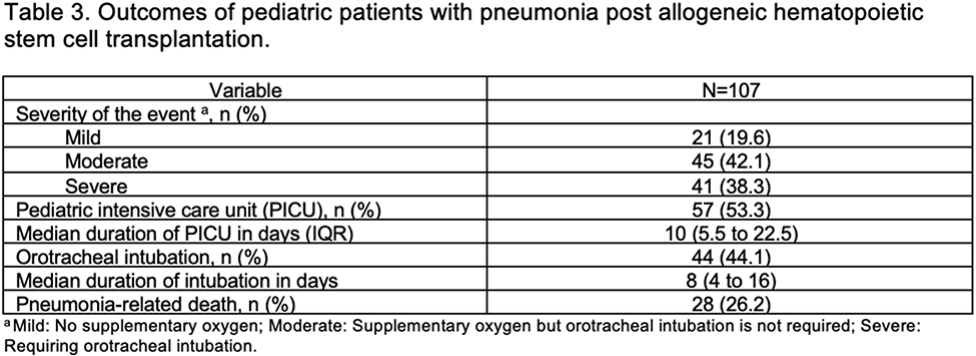

**Figure:**
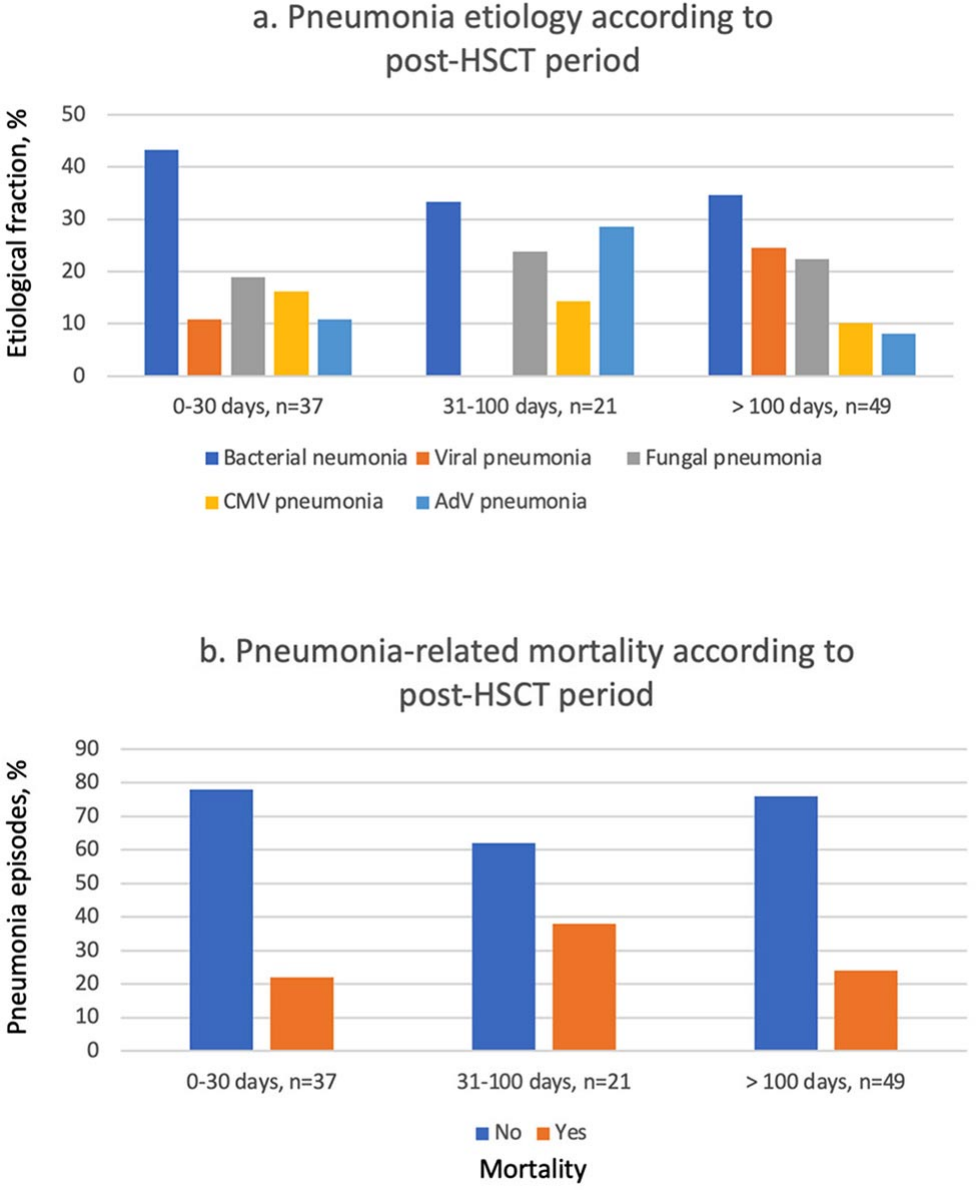

**Results:**

107 patients were included (median age 3.7 years; 57% male), with lymphoblastic leukemia as the most common HSCT indication. Pneumonia developed 83 days post-HSCT (median; IQR 22–237) (Table 1). Bacterial infections predominated, especially within the first 30 days post-transplant (Figure 1a). Pneumonia-related mortality occurred in 28 patients (26%), often between 30 and 100 days post-HSCT (Figure 1b). Mortality was associated with reduced-intensity conditioning (Table 1) and in those presenting with hypotension or hypoxemia (Table 2). Lower death risk was observed in patients presenting with cough or upper respiratory symptoms. Deceased patients were more often neutropenic and lymphopenic, frequently had adenovirus-associated pneumonia and showed certain radiographic features in X-ray, such as central and peripheral infiltrates, as well as diffuse opacities (Table 2). Fifty-three and 44% of patients required intensive care and mechanical ventilation, respectively (Table 3).

**Conclusion:**

Pneumonia post-HSCT is a major complication in children with a high mortality rate. This well characterized cohort revealed clinical, temporal, etiological, laboratory, and radiologic factors associated with mortality. Continued collaboration is essential to enhance the dataset, identify and validate independent predictors of mortality, and propose effective preventive strategies.

**Disclosures:**

Almudena Laris Gonzalez, MD, MSc, Astra Zeneca: Advisor/Consultant|GSK: Honoraria|Sanofi: Advisor/Consultant

